# Results of the Hematology Laboratory Survey: What has Changed in Eight Years?

**DOI:** 10.4274/tjh.2018.0065

**Published:** 2018-08-05

**Authors:** İlknur Kozanoğlu, Türkan Patıroğlu, Klara Dalva, Gülderen Yanıkkaya Demirel, Teoman Soysal, Muzaffer Demir

**Affiliations:** 1 Başkent University Dr. Turgut Noyan Practice and Research Hospital, Clinic of Hematology, Adana, Turkey; 2 Erciyes University Faculty of Medicine, Clinic of Hematology, Kayseri, Turkey; 3 Ankara University Faculty of Medicine, Clinic of Hematology, Ankara, Turkey; 4 Yeditepe University Faculty of Medicine, Clinic of Hematology, İstanbul, Turkey; 5 İstanbul University Cerrahpaşa Faculty of Medicine, Department of Hematology, İstanbul, Turkey; 6 Trakya University Faculty of Medicine, Department of Hematology, Edirne, Turkey

**Keywords:** Hematology laboratory, Survey, Turkish Society of Hematology and Laboratory Subcommittee

The Scientific Subcommittee on Laboratory Standards of the Turkish Society of Hematology (TSH) conducted two surveys, in 2009 and 2017, evaluating the tests, devices, and systems used in hematology laboratories (or other laboratories where hematological analyses are performed) in Turkey. The survey was shared online with TSH members as an informational message. Results from the 2017 survey were compared with those obtained in 2009 [1].

The survey was completed by 18 laboratories (14 university hospitals, 2 Ministry of Health education and research hospitals, 1 research institute, 1 private hospital) in 2009 and by 20 laboratories (12 university hospitals, 6 Ministry of Health hospitals, 2 foundation universities) in 2017 (Table 1).

In 2009, 11 (61%) laboratories were independent and 2 (11%) were part of a central laboratory. In 2017, 3 (15%) were independent and 12 (60%) were part of a central laboratory.

Regarding employed personnel, respondents in 2009 indicated that 24 medical doctors, 71 biologists, and 75 technicians worked in the laboratories. Respondents in 2017 indicated that 12 medical doctors, 12 biologists, and 16 technicians were employed.

In 2009, only three laboratories conducted internal quality control analyses for all tests. In 2017, internal quality control was conducted for all tests in seven laboratories, flow cytometry in two laboratories, coagulation in two laboratories, and electrophoresis in one laboratory. External quality control programs were utilized in 15 laboratories in 2009 and 9 in 2017. A written hematology laboratory manual was used by 13 (72.2%) and 11 (55%) laboratories in 2009 and 2017, respectively.

Performance of molecular studies, flow cytometry analyses, and minimal residual disease tests increased over the 8-year period. Additionally, 12 (60%) laboratories surveyed in 2017 had automation systems for peripheral blood smears, while none had automation systems in 2009.

In the 2017 survey, eight laboratories responded to the question “What are your expectations from the Laboratory Subcommittee?” Three respondents expressed their views on efforts to develop regulations pertaining to existing legislation, two indicated a desire for more active training, and three discussed efforts to prepare laboratory guidelines.

Hematology laboratories have not been defined in the Turkish Medical Laboratories Regulation (2010, 2013), which regulates procedures and principles regarding the planning, licensing, opening, regulating, classifying, monitoring, controlling, and terminating of activities of medical laboratories. This has led to the closure of many hematology laboratories and/or their inclusion into a central laboratory system.

Between 2009 and 2017, the number of personnel working in hematology laboratories in Turkey decreased. The hardware and infrastructure are in a position to match the developing technology, but not the standardization [2]. The TSH and the Scientific Subcommittee on Laboratory Standards are closely monitoring the current legislation and efforts are continuing to improve the existing legal situation.

## Figures and Tables

**Table 1:**
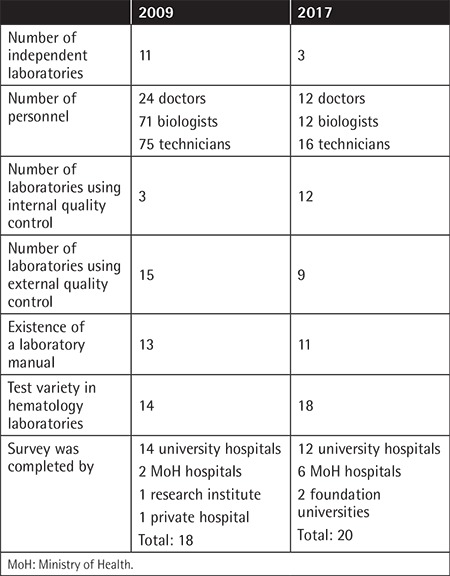
Changes of the hematology laboratories in Turkey between the 2009 and 2017 surveys.
